# Seroprevalence of brucellosis antibodies and associated risk factors among the hospitalized patient, Aleppo, Syria: a hospital-based cross-sectional study

**DOI:** 10.1097/MS9.0000000000001687

**Published:** 2024-01-05

**Authors:** Mohammed Abdulrazzak, Mohammed Moutaz Alshaghel, Rami Anadani, Muhammad Besher Shabouk, Moustafa Alhashemi, Fatima Breim, Khaled Ali Alahmad, Mohammed Alabrash, Samer Haj Kadour

**Affiliations:** a Faculty of Medicine; b CME Office, Faculty of Medicine, University of Aleppo; c Department of Laboratory, Aleppo University Hospital, Aleppo, Syria

**Keywords:** 2-Mercaptoethanol, Aleppo, brucellosis, seroprevalence, Syria, Wright test

## Abstract

**Introduction and importance:**

Brucellosis is one of the most common infectious diseases in the world, especially in developing countries. Recent reports show that Syria is among the top ten countries where brucellosis is most prevalent. The purpose of this study is to estimate the seroprevalence of brucellosis antibodies among the hospitalized patients, in one of the largest hospitals in northern Syria.

**Materials and methods:**

A cross-sectional study was conducted among the hospitalized patients. The authors used a questionnaire to collect sociodemographic and brucellosis-related data from the patients. The authors also collected blood samples from these patients to be screened for brucellosis antibodies using Wright Coombs Agglutination and 2-mercaptoethanol tests, during the period from November 2021 and March 2022.

**Results:**

Among the 776 patients who were recruited in the study, the seroprevalence of brucellosis antibodies was 13.1% (*n*=776). The highest prevalence was among the female sex (16.7%, *n*=298), middle aged group 12–40 years (24.1%, *n*=116), and patients with history of brucellosis (30.1%, *n*=53). Among the positive samples, the findings of 2-mercaptoethanol tests show that (14.7%, *n*=102) were positive (presence of IgG Antibodies), and (75.5%, *n*=102) were negative.

**Conclusion:**

This study is the first to describe the epidemiology of brucellosis in northern Syria. It clearly shows high rates of positivity, which reflects immense challenges facing the public health sector in Syria. The best next step in light of this crisis is to raise awareness among population about brucellosis and its risk factor.

## Introduction

Highlights

- Brucellosis is one of the most common infectious diseases in the world, especially in developing countries. Recent reports show that Syria is among the top 10 countries where brucellosis is most prevalent.
- This study is the first to describe the epidemiology of brucellosis in northern Syria.
- It shows high rates of positivity, which reflects immense challenges facing the public health sector in Syria.

Brucellae are gram-negative coccobacillus bacteria that belong to the Brucellaceae family^1^. They are zoonotic, non-motile, non-spore-forming, and facultative intracellular microorganisms that grow slowly on blood agar^1,2^. Of the 12 different species of brucella, only four have been reported to infect humans: B. melitensis (the most virulent species contributing to the majority of human cases), B. canis, B. suis, and B. abortus.

It is transmitted mainly by sheep and goats either directly (via direct contact between humans and these animals) or indirectly (via consumption of contaminated animal products for example milk or cheese)^1^—that is why it is most commonly seen in rural areas and in developing countries (e.g. the Middle East)^1,3^. Transmission of brucellosis from person to person is quite rare but possible via sexual contact, placenta, breastfeeding, blood transfusion, or transplantation^4–6^.

Clinical features of brucellosis are not pathognomonic, as most of the clinical symptoms are non-specific^2^. The most common clinical presentations are arthralgia, sweating, fever, and backache^6^.

Laboratory diagnosis of brucellosis may include blood cultures, serology, and nucleic acid amplification tests (NAATs)^2^. Serological testing includes the detection of immune antibodies formed by the host against this microorganism. Hence, it is affected by the medical history of the patients, their exposure, and the status of their immune system^7^. Furthermore; the importance of serological studies comes from the fact that in endemic areas such as Syria, a large proportion of the infected population are asymptomatic and have a self-limiting disease^8^.

Brucellosis is the most prevalent bacterial zoonotic infection with half a million new cases every year^9^. The main problem with brucellosis is not its clinical severity—as it is usually not lethal—rather, it adds a real economic burden due to great losses in animal resources as well as the complicated treatment of brucellosis in humans^10^.

According to the most recent global brucellosis map in 2006, Syria had the highest annual incidence of brucellosis globally reaching a dangerous rate of 1603 cases per million annually^3^. Thus, our study aimed to discover the prevalence of brucellosis antibodies in randomly selected blood samples of random hospitalized patients, using Wright Coombs Agglutination Test as per The STROCSS 2021 Guideline^11^.

## Materials and methods

### Place of study

The study was conducted in one of the largest hospitals in northern Syria. The hospital is located in the centre of the city of Aleppo, the second largest governorate in Syria, and provides free general and specialized medical services for all patients from rural and urban areas.

### Study participants

Data collection was between November 2021 and March 2022, 3–4 days per week. Data were collected from random groups of patients admitted to the hospital in different departments. Only hospitalized patients with no suspicion of active brucellosis based on symptoms and medical examination were included. Outpatients and any patient was suspected of brucellosis were excluded from the study.

### Study design and samples

This study is a hospital-based cross-sectional study. A group of trained collaborators examined the seroprevalence of brucellosis and conducted interviews with the patients who were admitted to the hospital during the study period for different diagnoses. We used only blood samples that are already drawn and sent to the laboratory for routine tests earlier on the day of the interview. We included patients from Internal Medicine, General Surgery, Cardiovascular Surgery, Neurosurgery, and Paediatrics departments. Exclusion criteria were patients who refused to be interviewed and patients in departments other than those stated previously. We also excluded duplicate or incomplete interviews.

### Data collection

Data were collected using an interviewer-administered questionnaire preceded by laboratory tests. The questionnaire has two sections: The first section is about sociodemographic information (gender, marital status, education attainment, occupation), and the location\area where the participant comes from.

The second one contained questions about risk factors and history of chronic diseases. The questionnaire was developed using information obtained from similar studies, and tested before our study began. A pilot- study was conducted which involved 112 patients. The data obtained from the pilot study were used in order to improve the questionnaire. Laboratory tests performed on blood samples are the side agglutination brucella test followed by a 2-Mercaptoethanol (2-ME) test for serum-antibody recognition.

### Sample collection

Serological tests were performed on participants’ serum samples in the laboratory. The wright agglutination test was applied for all samples as a rapid plate test (RPT) then the 2-ME test was performed for positive samples only.

### Wright’s serological test

Wright’s serological test is a method that allows for the diagnosis of acute brucellosis through the use of serological techniques. This test quantitatively measures the level of antibodies present in the serum by observing the agglutination reaction of a suspension of Brucella, which has been killed by exposure to formaldehyde and heat. A negative test result is indicated by the absence of agglutination. On the other hand, a positive test result is determined by comparing the degree of cloudiness observed in the test serum with that seen in the titration control tube. If the antibody titre is equal to or greater than 1/80 (120 IU/ml), it indicates the presence of active brucellosis. Lower titres, such as 1/40 or 1/20, suggest a suspicion of brucellosis^12^.

### Serological test

#### Rapid plate test

All valid samples included in the study underwent this test using Cenogenic Brucella melitensis and suis 0.5% febrile antigens, and a drop of brucella antigen mixed with 80 μl of serum in a clean plate which makes 1/20 titre. We waited for the agglutination for 1–3 min. Positive samples showing any sign of agglutination were tested again with a smaller amount of serum in order to determine the final titre. As our study participants were not infected with brucella, high titres were not expected. Therefore, we began from 1/20 titre. After determining the final titre for each positive sample, the 2-ME test was performed to distinguish between the types of antibodies.

#### 2-Mercaptoethanol

**Figure 1:**
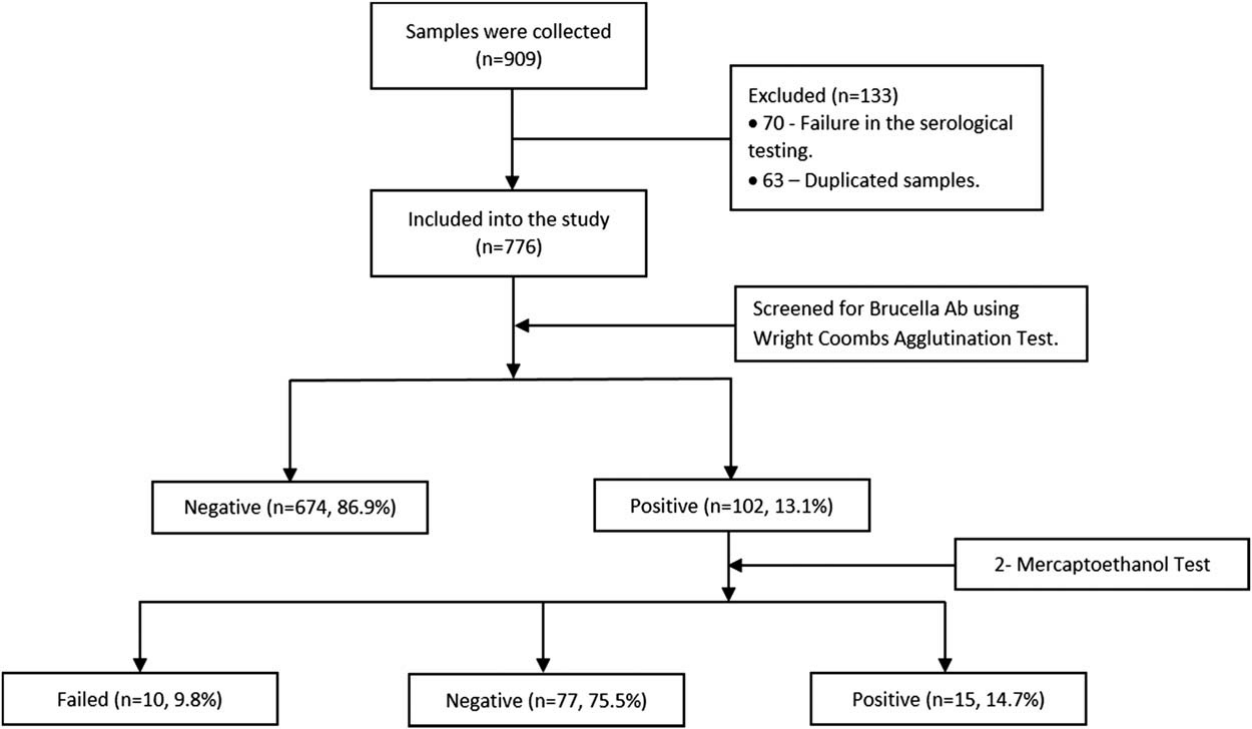
Summary of the outcomes of patient recruitment in the study.

Fifteen microlitres of the 2-ME solution was diluted in 1000 μl of water. The volume of the serum corresponding to the final titre of each positive sample was mixed with an equal volume of the 2-ME diluted solution. After 25–30 min, one drop of brucellosis antigen was mixed with the serum-2-ME on the same plate. Agglutination positivity refers to immunoglobulin G (IgG) antibodies present in the sample because 2-ME destroys the sulfur bonds in immunoglobulin M (IgM) antibodies (Fig. 1).

### Ethical considerations

Verbal informed consent was taken from each participant before the interview starts. Ethical Approval of this study was obtained from the Ethics committee of Faculty of Medicine.

### Statistical analysis

Interviews and test results were reported in paper forms, then filled in Google forms to organize the process and create a secure resource. Google forms were exported to an excel file in order to clean and prepare the data for statistical analysis using SPSS 25.0 (SPSS Inc.). Qualitative data were reported as frequency and percentage. The χ^2^ test and Odds ratio were applied to compare categorical variables. Bivariate data analysis was conducted to establish the risk factors associated with brucellosis in humans and odds ratios were obtained at 95% CI. A *P* value less than 0.05 was considered statistically significant in all tests. The variables that showed significance, *P*=0.05 at bivariate analysis were included in multivariable analysis.

## Results

### Randomly selected blood samples

A total of 909 random samples were collected from the Laboratory Department between November 2021 and February 2022. We excluded a total of 133 samples—70 samples due to failure in the serological testing process and 63 duplicate samples. We ended up with 776 samples.

The demography of the population can be seen in (Table 1). The majority of our population were older adults (40.3% were between the age of 40 and 65 years, and 27.2% were older than 65 years). Male patients composed 61.6% of the population and females composed 38.4%. Married people were more than double the single ones representing 63.0% of the population. As our sample from a public hospital providing free medical care, most of the population was from the lower socioeconomic class (52.2% uneducated and 74.7% unemployed). Patients from urban areas were 61.3% and patients from rural areas were 38.7% of the total population. Most of our patients have at least one diagnosed chronic disease (57.0%). The most frequently reported chronic diseases among the population were hypertension (34.4%) and diabetes (24.1%). It was observed that 24.7% of the population lived in close proximity to wild animals or had frequent interactions with them. Additionally, 81.3% of the individuals reported regular consumption of dairy products. A minority, comprising 6.8% of the respondents, had a history of Brucellosis, while 13.1% reported having contact with someone previously infected with Brucellosis.

**Table 1:** Summary of the sociodemographic characteristics of 767 study participants

	Total (*N*=776), n, (%)	Brucellosis positivity (*N*=102), *n*, (%)	*P*
Age groups			0.**001**
<12 years	136 (17.5)	5 (0.6)	
12–40 years	116 (14.9)	28 (3.6)	
40–65 years	313 (40.3)	43 (5.5)	
>65 years	211 (27.2)	26 (3.4)	
Sex			**0.018**
Male	478 (61.6)	52 (6.7)	
Female	298 (38.4)	50 (6.4)	
Marital status			0.275
Single	211 (27.2)	20 (2.6)	
Married	489 (63.0)	70 (9.0)	
Widowed	66 (8.5)	11 (1.4)	
Divorced	10 (1.3)	1 (0.1)	
Educational status			0.061
Uneducated	405 (52.2)	44 (5.7)	
Primary	200 (25.8)	37 (4.8)	
Mid-school	73 (9.4)	6 (0.8)	
Secondary	54 (7.0)	9 (1.2)	
College	44 (5.7)	6 (0.8)	
Place of Residency			0.732
Rural	300 (38.7)	41 (5.3)	
Urban	476 (61.3)	61 (7.9)	
Field of Work			0.293
Unemployed	580 (74.7)	70 (9.0)	
Low Risk	169 (21.8)	27 (3.5)	
High Risk	27 (3.5)	5 (0.6)	
Living near wild animals			0.664
No	584 (75.3)	75 (9.7)	
Yes	192 (24.7)	27 (3.5)	
Consuming milk products			0.798
No	145 (18.7)	20 (2.6)	
Yes	631 (81.3)	82 (10.6)	
History of Brucellosis			**0.001**
No	723 (93.2)	86 (11.1)	
Yes	53 (6.8)	16 (2.1)	
Contact with someone infected of Brucellosis			0.079
No	674 (86.9)	83 (10.7)	
Yes	102 (13.1)	91 (2.4)	
Chronic diseases			0.293
Negative chronic disease	334 (43.0)	39 (5.0)	
Posititve chronic disease	442 (57.0)	63 (8.1)	
Hypertension	267 (34.4)		
Diabetes	187 (24.1)		
Heart Diseases	73 (9.4)		
Chronic Kidney Disease	39 (5.0)		
Asthma	32 (4.1)		

Of the total 776 samples, 102 samples tested positive for Brucellosis (13.1%), and 674 tested negative (86.9%) (Table 2). Positive samples were further investigated to determine the serological concentrations of their antibodies—7.6% of the positive samples tested positive only at 1/20, 4.4% at 1/40, 0.8% at 1/80, and 0.4% at 1/160 (Table 2). Finally, a solution of 2-ME was added to the positive samples. Of the 102 positive samples, only 15 samples showed the presence of IgG Antibodies after the addition of 2-ME, 77 showed the presence of IgM Antibodies, and 10 samples were excluded due to failure in the process (Table 2).

**Table 2:** Serological-tests results

Wright tests	
	Number (*N*=776) [Percentage (100%)], *n* (%)
Negative	674 (86.9)
Positive	102 (13.1)
1/20	59 (7.6)
1/40	34 (4.4)
1/80	6 (0.8)
1/160	3 (0.4)
2-mercaptoethanol tests	
	Number (*N*=102) [Percentage (100%)], *n* (%)
2-ME negative	77 (75.5)
2-ME positive	15 (14.7)
Failed	10 (9.8)

2-ME, 2-mercaptoethanol.

There was a significant difference in positivity rates between males and females—10.9% and 16.8%, respectively (*P*=0.018). The highest percentage of positive results was found in the age group 12–40 years (24.1%) followed by the age group 40–65 years (13.7%). Positive rates were higher in rural areas in the age group 12–40 years (*P*=0.023). In the same age group, people living near wild animals showed positive Wright tests more than others. More than one-third of the patients who had a history of brucellosis tested positive for the antibodies (*P*=0.001). Only in the age group 40–65 years, there was an obvious correlation between recorded contact history with Brucellosis patients and positivity (*P*=0.037) (Table 3). We did not find any correlation between positivity and field of work or dairy products consumption (*P*>0.05).

**Table 3:** Comparison of positivity among age groups

	<12 (*N*=136), *n*, (%)	Brucellosis positivity, *P* value, *n*, (%)	12–40 (*N*=116), *n*, (%)	Brucellosis positivity, *P* value, *n*, (%)	40–65 (*N*=313), *n*, (%)	Brucellosis positivity, *P* value, *n*, (%)	65<(*N*=211), *n*, (%)	Brucellosis positivity, *P* value, *n*, (%)
Sex		0.851		0.730		0.112		0.194
Male	87 (64.0)	3 (2.2)	53 (45.7)	12 (10.3)	208 (66.5)	24 (7.7)	130 (61.6)	13 (6.2)
Female	49 (36.0)	2 (1.5)	63 (54.3)	16 (13.8)	105 (33.5)	19 (6.1)	81 (38.4)	13 (6.2)
Marital status				0.805		0.980		0.257
Single	136 (100)	5 (3.7)	53 (45.7)	13 (11.2)	15 (4.8)	2 (0.6)	7 (3.3)	0
Married	0	0	60 (51.7)	15 (12.9)	272 (86.9)	38 (12.1)	157 (74.4)	17 (8.1)
Widowed	0	0	2 (1.7)	0	19 (6.1)	2 (0.6)	45 (21.3)	9 (4.3)
Divorced	0	0	1 (0.9)	0	7 (2.2)	1 (0.3)	2 (0.9)	0
Educational status		**0.01**		0.956		0.757		0.488
Uneducated	115 (84.6)	2 (1.5)	40 (34.5)	10 (8.6)	140 (44.7)	19 (6.1)	110 (52.1)	13 (6.2)
Primary	19 (14.0)	3 (2.2)	45 (38.8)	12 (10.3)	77 (24.6)	12 (3.8)	59 (28.0)	10 (4.7)
Mid-school	2 (1.5)	0	17 (14.7)	3 (2.6)	40 (12.8)	3 (1.0)	14 (6.6)	0
Secondary	0	0	4 (3.4)	1 (0.9)	35 (11.2)	6 (1.9)	15 (7.1)	2 (0.9)
College	0	0	10 (8.6)	2 (1.7)	21 (6.7)	3 (1.0)	13 (6.2)	1 (0.5)
Place of residency		0.142		**0.023**		0.371		0.130
Rural	71 (52.2)	1 (0.7)	49 (42.2)	17 (14.7)	112 (35.8)	18 (5.8)	68 (32.2)	5 (2.4)
Urban	65 (47.8)	4 (2.9)	67 (57.8)	11 (9.5)	201 (64.2)	25 (8.0)	143 (67.8)	21 (10.0)
Field of work		0.845		0.051		0.968		0.301
Unemployed	135 (99.3)	5 (3.7)	78 (67.2)	18 (15.5)	179 (57.2)	25 (8.0)	188 (89.1)	22 (10.4)
Low risk	0	0	34 (29.3)	7 (6.0)	117 (37.4)	16 (5.1)	18 (8.5)	4 (1.9)
High risk	1 (0.7)	0	4 (3.4)	3 (2.6)	17 (5.4)	2 (0.6)	5 (2.4)	0
Living near wild animals		0.447		**0.031**		0.586		0.144
No	87 (64.0)	4 (2.9)	81 (69.8)	15 (12.9)	243 (77.6)	32 (10.2)	173 (82.0)	24 (11.4)
Yes	49 (36.0)	1 (0.7)	35 (30.2)	13 (11.2)	70 (22.4)	11 (3.5)	38 (18.0)	2 (0.9)
Consuming milk products		0.064		0.212		0.187		0.612
No	54 (39.7)	0	20 (17.2)	7 (6.0)	45 (14.4)	9 (2.9)	26 (12.3)	4 (1.9)
Yes	82 (60.3)	5 (3.7)	96 (82.8)	21 (18.1)	268 (85.6)	34 (10.9)	185 (87.7)	22 (10.4)
History of brucellosis		0.845		0.138		**0.001**		0.225
No	135 (99.3)	5 (3.7)	107 (92.2)	24 (20.7)	280 (89.5)	31 (9.9)	201 (95.3)	26 (12.3)
Yes	1 (0.7)	0	9 (7.8)	4 (3.4)	33 (10.5)	12 (3.8)	10 (4.7)	0
Contact with someone infected of brucellosis		0.560		0.280		**0.037**		0.484
No	120 (88.2)	4 (2.9)	102 (87.9)	23 (19.8)	266 (85.0)	32 (10.2)	186 (88.2)	24 (11.4)
Yes	16 (11.8)	1 (0.7)	14 (12.1)	5 (4.3)	47 (15.0)	11 (3.5)	25 (11.8)	2 (0.9)
Chronic diseases		0.299		0.845		0.397		0.119
Negative chronic disease	107 (78.7)	3 (2.2)	64 (55.2)	15 (12.9)	113 (36.1)	18 (5.8)	50 (23.7)	3 (1.4)
Positive chronic disease	29 (21.3)	2 (1.5)	52 (44.8)	13 (11.2)	200 (63.9)	25 (8.0)	161 (76.3)	23 (10.9)

## Discussion

This study is the first to describe the epidemiology of brucellosis in northern Syria. It shows that the prevalence of brucellosis antibodies among the hospitalized patients in our sample is 13.1%. It is close to hospital-based studies done in Uganda that reported prevalence of 13.3%, and 14.9%^13,14^, and lower than Prevalence of brucellosis among patients attending Wau Hospital in South Sudan 23.3%^15^.

Brucellosis is transmitted by exposure to infected animals (for example: cows, sheep, goats, and pigs) or their products. Certain occupations such as farmers, shepherds, veterinarians and workers at slaughterhouses are well known to be at higher risk due to increased exposure^16^.

Brucella is an intracellular organism, which explains the chronicity of the disease and the difficulty of treating it. The bacteria are quickly phagocytosed by mature macrophages, where they are capable of surviving and replicating^17^. The chronic nature of the disease enables the bacteria to modulate the immune system and invade a wide host of tissues, causing extensive damage to multiple organ systems. Chronic brucellosis can cause encephalitis, meningitis, endocarditis, arthritis and orchitis, among many other illnesses^14,15^. Multiple antibiotic treatments are available, however the misinterpretation of the nonspecific symptoms can delay the diagnosis and undermine the efficacy of the treatment^18^.

Serological tests play an essential role in the diagnosis and screening of brucellosis^19^. They detect the antibodies formed against the bacteria, including IgM, and IgG^20^. IgM levels start to increase in the first week of infection, followed by increased levels of IgG in the second week. Both antibodies peak after 1 month of infection, but, the levels of IgG antibodies remain lower than IgM at all times of infection, and decline quickly after treatment^19,21^. On the other hand, IgG antibodies may remain positive for months, even after treatment. It is worth noting that the immune response to brucellosis varies widely among people^20^.

Some countries (such as Australia, Germany, Finland and the UK) have controlled the disease, however it still remains prevalent in many regions such as the Middle East, Latin America, and central and eastern Asia^22^. Syria is among the top ten countries with the highest incidence^23^. Underdiagnosis and undertreatment due to lack of adequate awareness and availability of treatment further increases the socioeconomic burden of this disease^24^.

Vaccines and test-and-slaughter are among the commonest and most effective control measures. The difference in efficacy has been controversial and many studies have been made to compare between them. Zinsstag has demonstrated that vaccines can control brucellosis however they cannot fully eradicate the disease. It has been shown that the vaccines may not be so effective in preventing infection. Therefore, the combination of the methods seems to be superior to one of them alone^25,26^. However, the use of low-quality vaccines of reduced dosages may give a false sense of comfort, for example, in Jordan where they vaccinated using a lowered dose of an uncommon vaccine^27,28^. Australia, New Zealand, and the United States are fully free of brucellosis and their experiences can guide other countries. They implemented vaccination and test-and-slaughter programs, in addition to other control measures^25^.

There must be a cohesive and integrated system that’s specialized in the surveillance of the disease. Without these crucial data, any control efforts would be ineffective^29,30^.

One might ask: why are the majority of countries failing to successfully control the disease despite the available vaccines and useful experiences?

Well in the case of Syria, and most Middle Eastern and other low-income countries, the lack of necessary funds is a main obstacle. Other major hindrances include the sociopolitical unrest that has and still is devastating many of Middle Eastern countries makes it difficult for governments to implement, follow and enforce adequate control programs; the lack of cooperation of neighbouring countries; and the continuous war in these regions has led to the emigration of a large percentage of specialists^27,31^. Also, many governments of endemic countries (such as China), refuse to experiment with newer vaccines and remain working with the older one, which hinders the process of developing and testing better vaccines^32^.

It would be naïve to say that the road to controlling brucellosis worldwide is straightforward. The oversimplification of this crisis is detrimental, and all concerned agencies and governments must know the seriousness of the problem. Vaccines and other control measures are effective; however, these efforts take many decades to show meaningful results, and most developing countries do not possess the capabilities nor the motivation to pursue decade-long programs.

One Health approach is how all concerned parties must think. This is an approach developed to gain an in-depth understanding of zoonotic infectious diseases, recognize the major factors at play, and plan appropriately to modify these factors. This approach emphasizes the importance of having a panoramic view where controlling one aspect of the problem is not enough to gain long-term control of the disease. One Health identifies four key elements that must be taken into consideration when dealing with zoonotic disease: the geographical, the ecological, the human activities, and the food-agricultural components^33^.

One clear example of how programs suffer due to lack of One Health implementation is Greece. The government implemented better sanitary measures and encouraged the pasteurization of milk, which led to marked decrease in infection rate, especially foodborne brucellosis. The positive results tricked the government into thinking that controlling the disease has become easily feasible, and they started a vaccination campaign. However, in the end, they had to halt vaccinations and start a test-and-slaughter campaign due to the increase in occupational infections. The government failed to see the panoramic picture where many behaviours and factors play a role in foodborne and occupational infection^34,35^.

In our study, Brucella antibody positivity rates were higher during the summer. The reason behind this is not straightforward, as the relationship between infectious diseases and climate change are multifactorial. Lie and colleagues proposed some reasons behind this increase^36^. Warmer temperatures and increased exposure to sunlight might enhance the transmissibility of the disease. Also, it is well known that during the summer, the livestock sector is at its most active period. This gives yet another strong example why adopting the One Health approach is crucial in understanding all factors contributing to this crisis.

One major aspect that must be addressed is awareness. Zhang *et al*.^29^ carried out a systematic review of awareness and found out that of signs and symptoms of human brucellosis in the available studies was 41.6%, while awareness of animal brucellosis was 24.8% and awareness of available vaccines was 26.1%. This demonstrates the catastrophic lack of awareness regarding this disease in all group of the community, which is reflected as increase difficulty of implementing adequate control measures.

In Syria, where a significant proportion of the people live in the rural areas, awareness becomes extremely vital. The lack of awareness leads to negligence and increased rates of occupational and foodborne illnesses, as well as delay of seeking medical help because of not knowing the symptoms and the serious nature of the disease^23,29^.

Many international and local volunteer groups have noticed this problem. It has been found that in Asia and Africa a significant portion of the population lack adequate awareness and that reflects in unhygienic behaviour. Also, in these rural areas, friends and family members are the main sources of information, therefore mass media-based or social media campaigns are of no use in reaching these communities. What must be done is launching awareness campaigns to raise awareness, and allowing the correct information to spread between them^29^. In Syria, there are many awareness campaigns, some aimed at farmers, and other to women who are responsible for practicing hygiene to protect the families.

The disease has a significant economic burden and, it’s far more economically sound to tackle this disease rather than neglecting it. McDermot et al noted that most economical studies that analyses the burden of brucellosis are made in the developed countries, whereas the developing countries are the ones that are being harmed more^37^. The devastating losses of livestock and costs of diagnosis and treatment pose an immense burden on the developing countries. In Syria, where the healthcare system is already under immense pressure and farmers and livestock holders are suffering of the fragile economy, brucellosis comprises a serious but severely neglected economical issue.

There should be incentive for the governments to focus more on this crisis. Half of the countries in the middle east, including Syria, have a per capita gross domestic product (GDP) less than the average of the world. Also, the fact that Syria has been and still is suffering of a major socioeconomically distress evades no one. Therefore, international aid should be targeting countries like Syria, in need of many kinds of support to tackle the crisis^27,38^.

One Health remains the best solution for all problems that are hindering the process of controlling brucellosis. A multidisciplinary system that takes into consideration all aspects contributing to the problem, ensures comprehensive planning and international cooperation, and overseas the applications of these plans. More should be done to encourage governments and NGOs to adopt One Health interventions.

In the end, we must point to the limitations of our study. We could not use ELISA due to the scarcity of the available resources and ELISA is reserved for only clinical diagnostic purposes. Also, we took our sample from inpatient wards, therefore all outpatient admissions were excluded. These restrictions we imposed due to the additional, overwhelming pressure that the COVID-19 pandemic put on our already fragile healthcare system.

Our study is not by any means sufficient to draw final conclusions. However, we find that it sheds much-needed light on the state of this crisis, especially in low-income countries such as Syria. We hope that this study drives more researchers and organizations to further the frontier of our knowledge of this disease, taking into the consideration the intricacies that can be found in countries suffering from a prolonged unrest.

## Conclusion

Brucellosis is one of the most common public health problems in Syria. Our study investigated the prevalence of serum antibodies in an uninfected sample from one of the largest hospitals in Syria. The results reflect how much individuals are surrounded by risk factors. Comprehensive health awareness and cooperation between non-governmental organizations and the staff of the Directorate of Health is the best way to eliminate risk factors and prevent disease development.

## Ethics approval and consent to participate

The study was approved by the Ethics committee of Faculty of Medicine.

## Consent for publication

All authors provide consent for publication.

## Sources of funding

There are no funding sources.

## Authors contribution

M.A. and M.M.A. were involved in the design of the study. M.M.A., M.A., M.A., F.B., K.A.A.A., and M.A. made interviews and collected data from patients. M.M.A., M.A., M.A., F.B., K.A.A.A., and M.A. conducted laboratory tests in the central laboratory of the hospital. M.M.A. performed the data analysis. M.B.S., R.A., M.A., and M.M.A. wrote the original draft. M.B.S., R.A., and M.A. reviewed and edited the final edition of the paper. S.H.K. supervised the laboratory process and is the senior author. All authors read and approved the final version of the manuscript.

## Conflicts of interest disclosure

The authors declare that they have no competing interests.

## Research registration unique identifying number (UIN)

Research Registry researchregistry9685 https://researchregistry.knack.com/researchregistry#home/registrationdetails/654f7bb34cbf4700272de835/.

## Guarantor

All authors.

## Availability of data and material

The datasets generated and analyzed during the current study are not publicly available.

## Provenance and peer review

Not commissioned, externally peer-reviewed.
